# Evaluation of Porcine Versus Human Mesenchymal Stromal Cells From Three Distinct Donor Locations for Cytotherapy

**DOI:** 10.3389/fimmu.2020.00826

**Published:** 2020-05-06

**Authors:** Riccardo Schweizer, Matthias Waldner, Sinan Oksuz, Wensheng Zhang, Chiaki Komatsu, Jan A. Plock, Vijay S. Gorantla, Mario G. Solari, Lauren Kokai, Kacey G. Marra, J. Peter Rubin

**Affiliations:** ^1^Department of Plastic Surgery, University of Pittsburgh Medical Center, Pittsburgh, PA, United States; ^2^McGowan Institute for Regenerative Medicine, University of Pittsburgh, Pittsburgh, PA, United States; ^3^Department of Plastic Surgery and Hand Surgery, University Hospital Zurich, Zurich, Switzerland; ^4^Department of Plastic, Reconstructive and Aesthetic Surgery, Gulhane Military Medical Academy, Ankara, Turkey; ^5^Department of Bioengineering, University of Pittsburgh, Pittsburgh, PA, United States

**Keywords:** adipose-derived stromal cells, bone marrow stromal cells, endothelial growth medium, immunomodulation, omentum majus, immunosupressants, mixed lymphocyte reaction, transplantation

## Abstract

**Background:** Mesenchymal stromal cell (MSC)-based cytotherapies fuel the hope for reduction of chronic systemic immunosuppression in allotransplantation, and our group has previously shown this capability for both swine and human cells. MSCs harvested from distinct anatomical locations may have different behavior and lead to different outcomes in both preclinical research and human trials. To provide an effective reference for cell therapy studies, we compared human and porcine MSCs from omental fat (O-ASC), subcutaneous fat (SC-ASC) and bone marrow (BM-MSC) under rapid culture expansion with endothelial growth medium (EGM).

**Methods:** MSCs isolated from pigs and deceased human organ donors were compared for yield, viability, cell size, population doubling times (PDT), surface marker expression and differentiation potential after rapid expansion with EGM. Immunosuppressant toxicity on MSCs was investigated *in vitro* for four different standard immunosuppressive drugs. Immunomodulatory function was compared in mixed lymphocyte reaction assays (MLR) with/without immunosuppressive drug influence.

**Results:** Human and porcine omental fat yielded significantly higher cell numbers than subcutaneous fat. Initial PDT was significantly shorter in ASCs than BM-MSCs and similar thereafter. Viability was reduced in BM-MSCs. Porcine MSCs were positive for CD29, CD44, CD90, while human MSCs expressed CD73, CD90 and CD105. All demonstrated confirmed adipogenic differentiation capacity. Cell sizes were comparable between groups and were slightly larger in human cells. Rapamycin revealed slight, mycophenolic acid strong and significant dose-dependent toxicity on viability/proliferation of almost all MSCs at therapeutic concentrations. No relevant toxicity was found for Tacrolimus and Cyclosporin A. Immunomodulatory function was dose-dependent and similar between groups. Immunosuppressants had no significant adverse effect on MSC immunomodulatory function.

**Discussion:** MSCs from different harvest locations and donor species differ in terms of isolation yields, viability, PDT, and size. We did not detect relevant differences in immunomodulatory function with or without the presence of immunosuppressants. Human and pig O-ASC, SC-ASC and BM-MSC share similar immunomodulatory function *in vitro* and warrant confirmation in large animal studies. These findings should be considered in preclinical and clinical MSC applications.

## Introduction

Mesenchymal stem cells (MSCs) are well-known for their beneficial potential in a variety of conditions since discovery of their multi-facetted ability to proliferate, differentiate, heal, regenerate and modulate (1). Due to their paracrine immunomodulatory function (2–4), different MSC types are increasingly used in transplantation as cell adjuncts paired to conditioning and maintenance regimens in an attempt to reduce the burdens of immunosuppression after allotransplantation and promote durable graft tolerance (5–7): intensive preclinical and clinical research is carried out to establish such new successful protocols in small and large animals.

Inherent advantages of ASCs over their bone-marrow counterpart have shifted the focus accordingly (8, 9). Beside the abundance and ease of access of fat depots in patients to harvest the cells from, ASCs are also supposed to be superior in terms of immunomodulatory function (8). Clearly, MSCs from distinct anatomical sources can potentially differ significantly in proliferative, differentiation and immunomodulatory capacity, to name a few, which has to be considered while performing preclinical, but also clinical studies including cell therapies (10–15).

Not only the anatomical cell origin is of relevance, but also the species may have impact on the functionality of MSCs: while porcine and human MSCs seem to possess similar characteristics suggesting that results can be extrapolated from preclinical studies and be applied to clinical protocols (16), other studies suggest different behavior of porcine MSCs compared to human cells (17).

Cell-based therapies for tolerance induction in transplantation are usually applied in conjunction with immunosuppressants employed as conditioning or maintenance medication, and these may indeed have an influence on viability and function of the applied cells. It has been shown previously that drugs such as mycophenolic acid (MPA) and rapamycin (Rapa) can disturb viability and proliferation of MSCs (18), but on the other hand also act synergistically as found with cyclosporin A (CsA) (19, 20).

Here, we aimed at directly comparing porcine MSCs harvested from three distinct anatomical locations head-to-head to their counterpart of human origin *in vitro* in terms of isolation yields, proliferation, immunosuppressive function, and susceptibility to different immunosuppressive agents, using a rapid expansion culture strategy including endothelial growth factor 2 (EGM-2) medium.

## Materials and Methods

### Donors and Tissue Harvesting

#### Animals

The cells were isolated from domestic Yorkshire pigs post-mortem (*n* = 7). The animals were euthanized by means of lethal pentobarbital injections and placed supine on an operating table. The isolation process was performed in a sterile fashion and the skin was scrubbed with betadine solution three times prior to skin incision. After an inguinal skin incision, all the subcutaneous inguinal fat was excised and placed in sterile containers. The tissue was irrigated with Ringer lactate to avoid any drying. Afterwards, a median laparotomy was performed and the whole omentum majus exposed and excised, then placed in a sterile container irrigated with Ringers lactate. Afterwards, the hind limb long-bones were harvested and cut-open at one end with an oscillating saw. The bone marrow was then flushed with RPMI-1640 with L-Glutamine (Fisher Scientific) directly in sterile containers. Data regarding isolation summarized in Table 1. The tissues were then immediately transferred to the cell isolation lab for further processing.

**Table 1:** Isolation data.

**Age (months)**	**Weights (kg)**	**SC fat tissue (ml)**	**O fat tissue (ml)**	**BM (# of long bones)**	**SC-SVF **(x10∧6)****	**O-SVF **(x10∧6)****	**BM-“SVF” **(x10∧6)****
**PORCINE DONORS (*****n*** **=** **7)**							
3.8 ± 1.0	49.43 ± 11.3	112.9 ± 36.4	44.71 ± 7.3	4 ± 0.0	8.9 ± 8.8	19.4 ± 17.0	13.8 ± 1.6
**HUMAN DONORS (*****n*** **=** **6)**							
45.1 ± 13.8	88.5 ± 18.6	115 ± 45	110 ± 43.6	35.5 ± 15	8.9 ± 4.4	30.7 ± 15	12.4 ± 5.2

*Tissue weights and cell numbers at isolation*.

#### Human Donors

Deceased tissue donors were referred after informed consent by the Center for Organ Recovery and Education (CORE). All tissue donors (*n* = 6) were brain-dead cadaveric solid organ donors and de-identified. Inclusion criteria were 18–65 years of age male and female subjects. Exclusion criteria were the presence of hepatitis B, C, or HIV, sepsis/positive serology results. Adipose tissue from abdominal subcutaneous fat and omental fat (300–500 g) was excised under sterile conditions after solid organ retrieval. Bone marrow (30 mL) was aspirated from the iliac crest using an 11-G J-style aspiration kit (DePuy Synthes, Procure™). Data regarding isolation summarized in Table 1. Sampling was approved by the Committee for Oversight of Research and Clinical Training Involving Descents (CORID No. 475).

### Cell Isolation

#### Porcine

For isolation of SC-ASC and O-ASC, the tissues were minced with sterile scissors and handled with sterile forceps under a laminar flow hood until a relatively homogenous fat mass was obtained. The tissues were distributed into 50 mL conical tubes at 5 mL aliquots and 35 mL of sterile enzymatic solution added. The enzymatic solution was composed of type II collagenase (Worthington Biochemical Corp, Lakewood, NJ, USA), Proteinase K (Sigma-Aldrich) and Hanks' balanced saline solution (HBSS; Fisher Scientific) (for 100 mL of harvested fat: 1.4 g collagenase and 175 mg proteinase in 700 mL HBSS). The tubes were placed in a shaking water bath at 37°C for 90 min. Next, the digestate was filtered through 12-ply sterile gauze that had been unfolded twice (final gauze filter was 3-ply). The tubes were centrifuged at 1,000 rpm for 10 min. at room temperature (RT) and supernatant discarded. 10 mL red blood cell (RBC) lysis buffer (NH_4_Cl, A-0171; KHCO_3;_ P-7682; EDTA, E-5134, all from Sigma; pH adjusted to 7.4, solution with de-ionized water) was added to each tube and cell pellets were disrupted by pipetting up and down gently. Lysates from 4 tubes were combined into 50 mL tube yielded approximately 40 mL buffer per tube. Additional gauze filtration was used as needed. Tubes were centrifuged again at 1,000 rpm for 10 min. at RT and supernatant discarded. Cells were resuspended in endothelial growth factor 2 medium (EGM-2MV, Lonza, CC-3156 & CC-4147) supplemented with penicillin/streptomycin 1% (Invitrogen), gentamycin 1% (Invitrogen) and amphotericin B 1% (initial seeding only; Invitrogen), counted and either plated or immediately cryopreserved in freezing medium (DMSO10%/FBS90%). The cells were initially seeded at 10,000/cm^2^. After overnight incubation for cell attachment, non-adherent cells were removed using a PBS (Sigma-Aldrich) wash (culture incubator at 37°C, 5% CO2, 95–98% humidity). For BM-MSC isolation, the solution with the flushed bone marrow was added to an equal part of enzymatic digestion solution and placed in a shaking water bath at 37°C for 30 min. After that, the isolation process was identical to the above-mentioned ASC isolation. The cells were initially seeded at 100,000/cm^2^ and washed with PBS the day after. The cells were allowed to get to 80–90% confluence and then harvested by means of 0.25% trypsin (Corning). MSCs were always counted by an automated, calibrated counting machine (Countess machine and Countess® Cell Counting Chamber Slides, Invitrogen) and re-plated at 5,000 cells/cm^2^ in T175 or T75 flasks (Corning), 20 or 10 mL were added, respectively, to the flasks and changed thrice weekly. Amphotericin B was added during the first plating to avoid any fungal infection and then withdrawn at the first passaging.

#### Human

SC- and O-ASCs were isolated according to our established protocol (21). Briefly, adipose tissue was minced and digested in type II collagenase solution in a water bath at 37°C with gentle agitation for ~30 min. The digested tissue was then filtered through sterile gauze and centrifuged at 1,500 rpm for 10 min. The cell pellet was suspended in erythrocyte lysis buffer and centrifuged at 1,500 rpm for 10 min. The pellet was then suspended in EGM as described above and filtered through sterile gauze to eliminate any cellular debris. Further processing and culture were the same as for porcine cells.

BM-MSCs were isolated according to a protocol previously published by Wolfe et al. (22). Briefly bone marrow aspirate was diluted in Hanks balanced salt solution, gently overlaid on Ficoll Paque Plus (GE-Healthcare) and centrifuged at 1,800 g for 30 min. After collection of the cloudy layer the cells were re-suspended with Hanks Balanced Salt Solution and centrifuged again at 1,000 g for 10 min. The cell pellet was suspended in EGM and plated overnight. Medium was changed the day after, and the cell culturing continued as described above.

### Cell Surface Marker Analysis

Passage 2 to 3 MSCs were lifted by means of trypsin, counted and put in sterile Falcon tubes at aliquots of 1 × 10^6^ cells/tube at least. The following antibody panel was used for characterization of porcine cells: CD14 (AbD Serotec), CD29 (BD Pharmingen), CD31 (BD Biosciences), CD34 (Abcam + Goat anti-rabbit IgG (PE-cy5.5), Life Technologies), CD44 (Biolegend), CD45 (Genway Biotech), CD73 (Biolegend), CD90 (BD Biosciences), CD105 (Bioss Antibodies), CD146 (Genetex). For human cells, following antibodies were used CD45, CD90, CD105, CD73, CD235a, CD34 (BD Biosciences), CD31 (BioLegend), CD33, CD14 (Beckman Coulter), and CD146 (Miltenyi Biotec) (antibodies summarized in Tables 2, 3). The cells were stained with the antibodies singularly and according to manufacturer's protocol and were assessed using a BD LSRII flow cytometer, analyzing at least 20,000 events (Becton Dickinson, Franklin Lakes, NJ, USA). The obtained data was analyzed with FlowJo software (TreeStar Inc., Ashland, OR, USA).

**Table 2:** Porcine panel.

**Marker**	**Dye**	**Vendor**
CD14	FITC	AbD Serotec
CD29	Alexa Fluor® 647	BD Bioscience
CD31	PE	BD Bioscience
CD34	PE-cy5.5 Ig-G	Abcam/Life Technologies (Dye)^*^
CD44	Alexa Fluor 700	Biolengend
CD45	DyLight®680	Genway Biotech
CD73	Brilliant Violet 421	Biolegend
CD90	DyLight®755	BD Bioscience
CD105	PE-cy7	Bioss Antibodies
CD146	FITC	Genetex

*Antibodies for flow cytometry phenotyping of porcine cells*.

* *CD34 was conjugated with PE-cy5.5. All other antibodies already delivered conjugated to mentioned dye*.

**Table 3:** Human panel.

**Marker**	**Dye**	**Vendor**
CD14	PC5	Beckman coulter
CD31	PE-Cy7	Biolegend
CD33	PC5	Beckman coulter
CD34	AF700	BD bioscience
CD45	APC-Cy7	BD bioscience
CD73	PE	BD bioscience
CD90	APC	BD bioscience
CD105	FITC	BD bioscience
CD146	VioBlue	Miltenvi biotec
CD235a	PE-Cy5	BD bioscience

*Antibodies for flow cytometry phenotyping of human cells*.

*All antibodies already delivered conjugated to mentioned dye*.

### Viability and Proliferation

For all the cell lines in culture, cell numbers were recorded at each passage and re-seeded at 5,000 cells/cm^2^. Proliferation was assessed until passage 7. The population doubling time (PDT) was calculated using following equation: time/log_2_(harvested cells/seeded cells) (23). Viability was assessed by tryptan blue (Invitrogen) exclusion at each passage and expressed as percentage.

### Adipogenic Differentiation Assays

Mesenchymal stem cells derived from the same individual/animal were plated at passage 3 at a density of 40,000 cells/cm^2^ in 96-well plates using EGM. After 24 h, medium was replaced with adipogenic differentiation medium [STEMPRO® Adipogenesis Differentiation Kit (Invitrogen)] that was changed every 3–4 days over the course of 2 weeks. Control cells were cultured in regular EGM for 2 weeks that was changed every 3–4 days. After 2 weeks the cells were the washed with PBS and each well was filled with 200 μL PBS with addition of 5 μL Adipored® staining solution and incubated for 10 min. The fluorescence readout was performed using a microplate reader (Infinite® 200 PRO NanoQuant, Tecan). After readout, cells were imaged with bright-field microscopy.

### Mixed Lymphocyte Reaction Assays

Responder splenocytes were isolated from spleens of naïve pigs and human donors and were stimulated by phytohemagglutinin (PHA; Sigma-Aldrich). In the suppressor assay, 200,000 cells/well MSCs were added at different ratios to responder cells, namely at 1:4, 1:8, and 1:16, in 96-well plates with round bottom. Co-cultures were performed in triplicates until confluence (3–5 days) with RPMI-1640 medium supplemented with L-Glutamine. To assess splenocyte proliferation, the cells were pulsed with [3H]thymidine (1 mCi/well) for the final 8 h and [3H]thymidine incorporation was measured as counts per minute in a liquid scintillation counter (Perkin Elmer). A total of 6 and 3 experiments were performed for porcine and human MLRs, respectively. For MLR testing under influence of immunosuppressants, the MSCs were incubated with the different compounds for 36 h and then added to MLR as described above. Following, clinically relevant concentrations were used for these experiments: 10 ng/mL (Tac), rapamycin 10 ng/mL (Rapa), Cyclosporin A 250 ng/mL (CsA).

### Toxicity Assays

The following drugs were used (all Sigma-Aldrich, St. Louis, MO, USA): tacrolimus (T-049), rapamycin (S-015), cyclosporin A (30024), and mycophenolic acid (M3536; MPA). AlamarBlue (AbD Serotec) was quality-controlled as suggested by the manufacturer and optimal cell seeding density and incubation time determined. Positive and negative controls were performed. A stock solution was created for each drug and then serially diluted. 5000 cells/well were seeded in 96-well plates (Corning) and incubated with the drugs for 36 h. Experiments were performed in quadruplicates and passage 3 to 4 cells were used. Following serial dilutions were used: Tac and Rapa 0 – 2 - 10 – 50 – 250 - 1250 ng/ml, CsA 0 – 10 – 50 – 250 – 1250 - 6250 ng/ml, and MPA 0 – 0.2 – 1 – 5 – 25 – 125 ug/ml. Sole addition of PBS served as control. At the end of incubation, 20 μl AlamarBlue was added to each well and incubated for 5 h. After that, 200 μl of supernatant were transferred from each well to a new, black 96-well plate with clear flat bottom (Fisher Scientific) and directly assessed for fluorescence in a plate reader (Tecan Infinite M200 Pro). A total of 8 readings per well was performed and mean calculated.

### Cell Size Measurement

Plates with detached cells were imaged and under an Axiovert 25 microscope (Carl Zeiss Microscopy GmbH) as described previously (24). Calibration occurred with a glass hemocytometer. Cell sizes were calculated with ImageJ software (National Institute of Health, Bethesda, MD, USA). For that purpose, at least 100 cells/sample were measured and means calculated.

### Statistical Analysis

Prism 8.0 (GraphPad, San Francisco, CA, USA) was used for statistical analysis. Data are presented as means ± SD unless otherwise specified. Differences between the groups were assessed by unpaired *t*-test (two groups), or one-way/two-way ANOVA with Tukey post-test (multiple groups). *P* < 0.05 was considered statistically significant.

## Results

### Cell Isolation

**Figure 1:**
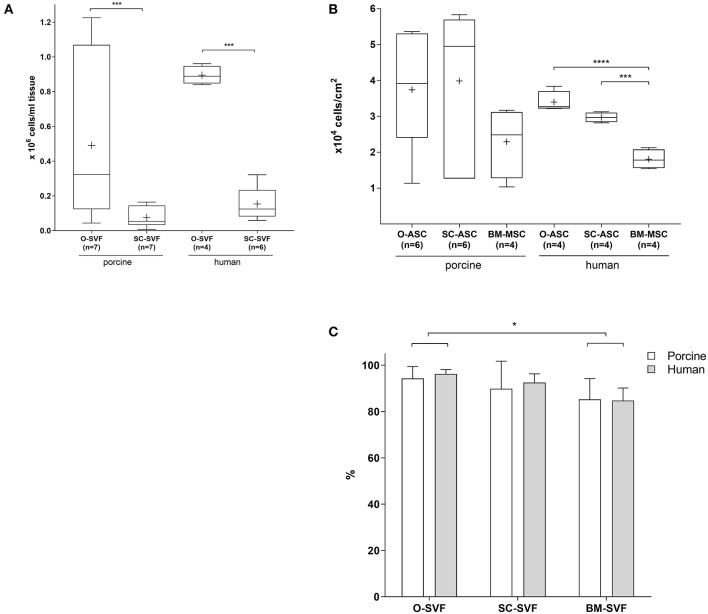
Cell isolation. **(A)** Cell yields after isolation following specific protocols for each cell type. The resulting cell population after isolation is the so called ≪stromal vascular fraction≫ (SVF). BM-MSC are not displayed since no precise tissue amount could be determined due to flushing. Expressed in number of cells per mL of harvested tissue. Cross = mean, whiskers = 5–95 percentile. **(B)** Cell yields after first passaging of plated cells at a density of 10,000 cells/cm^2^, passaged at 80–90% confluency. Expressed in number of cells per cm^2^. Cross = mean, whiskers = 5–95 percentile. **(C)** Viability of freshly isolated cells as determined by Tryptan blue exclusion. Expressed in viable cell % of total cells. Error bars = SD. Unpaired *t*-test. *p < 0.05, ***p < 0.001, ****p < 0.0001.

O-SVF of both porcine and human origin showed the highest yields per mL of tissue (pO-SVF 0.49 ± 0.4 and hO-SVF 0.894 ± 0.05 x10^6^/mL tissue), significantly higher than the SC counterpart (pSC-SVF 0.076 ± 0.05 and hSC-SVF 0.154 ± 0.09 × 10^6^/mL tissue; *p* < 0.001 vs. p/hO-SVF; Figure 1A). Since no exact amount of tissue could be retrieved for BM-MSC after flushing the long bones, isolation yields could not be calculated.

Cell yields after passaging the cultured SVF showed a trend for higher numbers in pO-ASC and pSC-ASC compared to BM-MSC (37,390 ± 16,268 and 39,859 ± 21,606 vs. pBM-MSC 22,917 ± 9,814 cells/cm^2^; n.s.). For human cells, however, there was a statistically significant higher proliferation in O-ASC and SC-ASC compared to their BM counterpart (hO-ASC 33,973 ± 2,936, and hSC-ASC 29,695 ± 1,336; *p* < 0.001 and *p* < 0.0001 vs. BM-MSC 18,070 ± 2,712 cells/cm^2^; Figure 1B).

While SVF viability upon isolation was comparable between O-SVF and SC-SVF from both porcine and human donors (pO-SVF 94.31 ± 5.15%, pSC-SVF 89.87 ± 11.89%, hO-SVF 96.25 ± 1.89%, hSC-SVF 92.5 ± 3.78%; p>0.05; Figure 1C), porcine and human BM-SVF revealed reduced viability (pBM-SVF 85.25 ± 8.97%, and hBM-SVF 84.75 ± 5.43%; *p* < 0.05 vs. p/hO-SVF).

### Cell Culture

**Figure 2:**
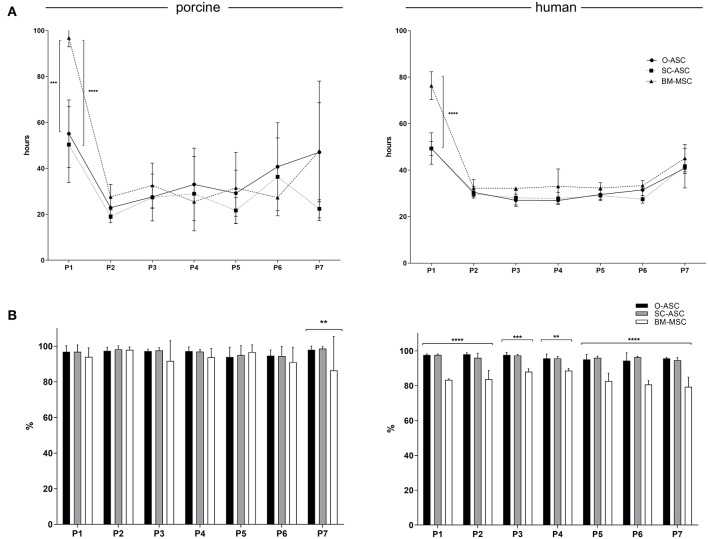
Cell culture. **(A)** Population doubling times (PDT) are shown for the three cell lines (O-ASC, SC-ASC, and BM-MSC) over 7 passages. Median ± interquartile range, expressed in hours. **(B)** The cell viability is displayed up to passage 7 for the three porcine and human cell lines (O-ASC, SC-ASC and BM-MSC) during culture with endothelial growth medium (EGM-2MV, Lonza). Determined by Tryptan blue exclusion. Cells lifted at 80–90% confluency. Expressed in %. Error bars = SD. One-way ANOVA with Tukey post-test. **p < 0.01, ***p < 0.001, ****p < 0.0001.

While first passage PDT was significantly longer in BM cells (pBM-MSC 96.80 ± 3.86 h, hBM-MSC 76.37 ± 5.98 h) compared to O- and SC-MSC (pO-ASC 55.1 ± 14.71 h, *p* < 0.001; pSC-ASC 50.36 ± 16.53h, *p* < 0.0001; hO-ASC 49.33 ± 6.78 h, *p* < 0.0001; hSC-ASC 49.3 ± 3.01h, *p* < 0.0001 vs. BM-MSC), the following PDT up to passage 7 was lower and comparable between the cells of different origin (Figure 2A). There was a trend for longer PDT for passage 7 in all groups beside pSC-ASC (pO-ASC 47.04 ± 21.59 h, pSC-ASC 22.40 ± 3.95 h, pBM-MSC 47.60 ± 30.40 h, hO-ASC 40.85 ± 8.49 h, hSC-ASC 41.70 ± 3.10 h, hBM-MSC 45.13 ± 5.84 h, n.s.).

Cell viability of porcine cells was comparable throughout passaging up to passage 6, whereas at passage 7 there was a decline in cell viability percent in BM-MSC group (86.50 ± 19.09% vs. pO-ASC 98.00 ± 2.09% and pSC-ASC 98.66 ± 1.15%; *p* < 0.01). For human-derived cells, BM-MSCs had significantly lower viability from passage 1 to 7 compared to O- and SC-ASCs (Figure 2B).

### Cell Surface Marker Analysis

**Figure 3:**
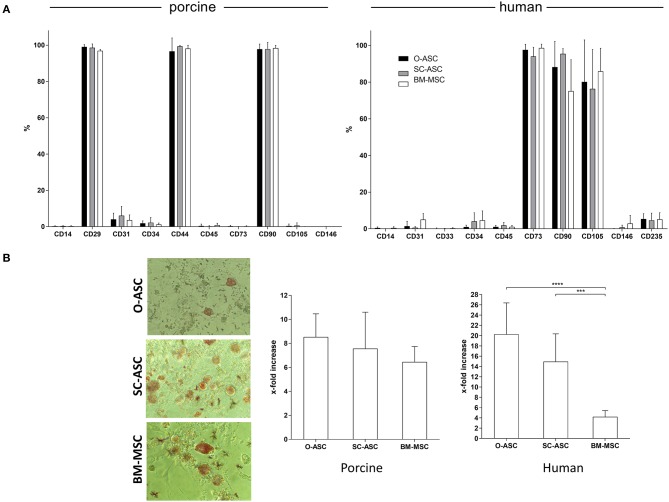
Cell surface marker phenotype and adipogenic differentiation. The three cell lines (O-ASC, SC-ASC, and BM-MSC) were analyzed for their surface marker phenotype by flow cytometry (**A**; Bencton FACS Aria). CD73 showed no cross-reaction. Porcine cells: O-ASC *n* = 9, SC-ASC *n* = 7, BM-MSC *n* = 4 runs. Human cells: O-ASC *n* = 4, SC-ASC *n* = 4, BM-MSC *n* = 3 runs. Expressed as %. Error bars = SD. Adipogenic differentiation assays were performed with passage 3 cells in triplicates. Differentiation was quantified by fluorescence after Adipored® staining. (**B** left panel) Representative microscopic pictures. (**B** right panel) Results expressed as x-fold increased intensity compared to controls. Error bars = SD. Two-way ANOVA with Tukey post-test. ***p < 0.001, ****p < 0.0001.

All cells of porcine origin showed high expression of CD29, CD44, and CD90, and negative for CD14, CD45, CD73, CD105, and CD146, while showing low expression for CD31 and CD34. Cells of human origin had a surface marker phenotype expressing high levels of CD73, CD90, and CD105 and negative, or low expression, for CD14, CD31, CD33, CD34, CD45, CD146, and CD235 (Figure 3A). hBM-MSC showed a non-significant lower expression of CD90 compared to hO-ASC and hSC-ASC (75.1 ± 17.09% vs. 88.22 ± 14.01% and 95.45 ± 2.80%).

### Adipogenic Differentiation

Swine-derived cells showed no relevant difference in terms of adipogenic differentiation potential between the cell types. X-fold fluorescence increase compared to control was 8.53 ± 1.93 for pO-ASC, 7.56 ± 3.03 for pSC-ASC and 6.45 ± 1.28 for pBM-MSC, respectively (n.s.; Figure 3B).

In contrast, in human cells there was a marked difference between the cell types: the highest adipogenic differentiation was in hO-ASC (20.27 ± 6.10 fold), followed by 14.95 ± 5.40 in hSC-ASC and 4.20 ± 1.20 in hBM-MSC (*p* < 0.0001 and *p* < 0.001 vs. O-ASC and SC-ASC; Figure 3B).

### Cell Size Measurement

**Figure 4:**
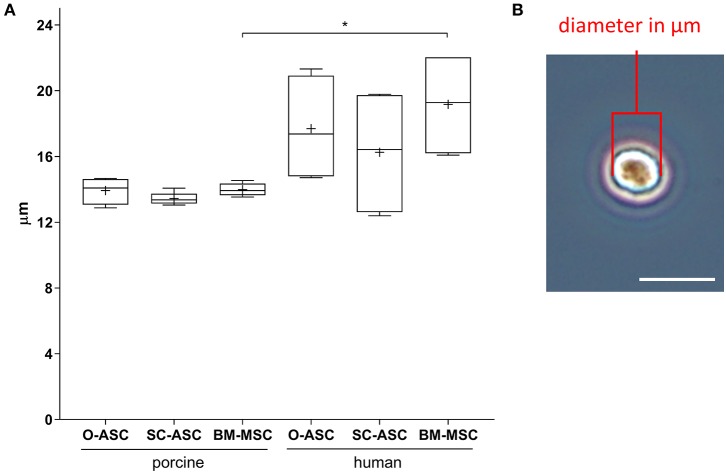
Cell size analysis. Diameters of lifted cells were measured in passage 2–3 cells for the three cell lines (O-ASC, SC-ASC, and BM-MSC). **(A)** Cell diameters expressed in μm. Error bars = SD. One-way ANOVA with Tukey post-test. *p < 0.05. **(B)** Representative microscopic image of one cell in the hemocytometer showing how diameters were manually measured with ImageJ (NIH). Bar = 20 μm.

Mean porcine cell sizes did not differ between the groups and were 13.93 ± 0.82 μm in pO-ASC, 13.45 ± 0.35 μm in pSC-ASC and 13.98 ± 0.38 μm in pBM-MSC. Compared to that, human cells showed slightly higher mean sizes, significant only for BM-MSCs (hO-ASC 17.70 ± 3.32 μm, hSC-ASC 16.26 ± 3.96 μm, hBM-MSC 19.17 ± 3.30 μm; hBM-MSC *p* < 0.05 vs. pBM-MSCs; Figure 4).

### Cell Immunomodulatory Function

**Figure 5:**
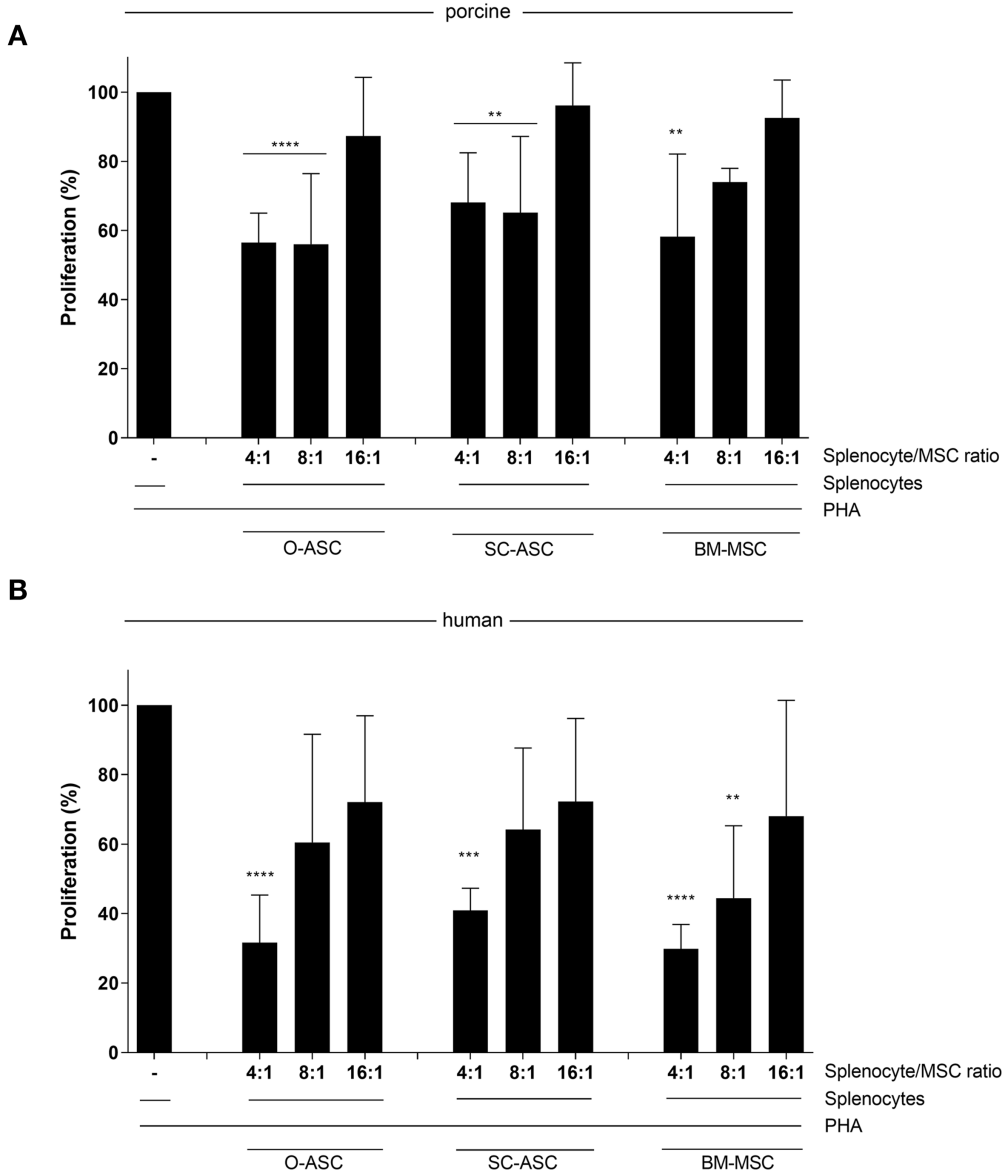
Immunosuppressive function. Mixed lymphocyte reaction assays (MLR) were performed to assess the immunomodulatory potential of the three cell lines (O-ASC, SC-ASC, and BM-MSC) head-to-head of both porcine **(A)** and human **(B)** origin. Yorkshire splenocytes were stimulated with PHA and co-cultured with the different cell lines for 3–5 days. Error bars = SD. Expressed as percentage proliferation in relation to positive control. Two-way ANOVA with Tukey post-test. **p < 0.01, ***p < 0.001, ****p < 0.0001 vs. control (splenocytes+PHA).

All three porcine MSCs demonstrated immunomodulatory function *in vitro* by reducing proliferation of allo-reactive splenocytes in MLR assays after addition at different ratios. O-ASC had the highest suppressive capacity, although not significant, compared to SC-ASC and BM-MSC, which had similar results. O-ASC (56.51 ± 8.51% at 4:1, 56.05 ± 20.38% at 8:1, *p* < 0.0001 vs. control) and SC-ASC (68.09 ± 14.38% and 65.12 ± 22.09%, for 4:1 and 8:1, respectively, *p* < 0.01 vs. control) had significant suppression at 4:1 and 8:1 ratios, while BM-MSCs only at 4:1 ratio (58.21 ± 23.88%, *p* < 0.01 vs. control). At splenocyte-to-MSC ratio of 16:1 there was no significant suppression in all three MSC lines (O-ASC 87.29 ± 16.97, SC-ASC 96.172 ± 12.31, BM-MSC 92.51 ± 10.98, *p* > 0.05 vs. control; Figure 5A).

Human cells, had a similar but stronger trend compared to porcine-derived counterparts, with all cell types being able to reduce allo-reactivity more consistently in a dose-dependent way; however, there was no significant difference between the groups (O-ASC 4:1 31.64% ± 13.69, 8:1 60.50% ± 31.11, 16:1 72.12% ± 24.79; SC-ASC 4:1 40.92% ± 6.39, 8:1 64.21% ± 23.44, 16:1 72.27% ± 23.91; BM-MSC 4:1 29.85% ± 7.06 8:1 44.43% ± 20.84 16:1 68.10% ± 33.27; all 4:1 *p* < 0.0001, O-ASC 8:1 *p* < 0.05 and BM-MSC 8:1 *p* < 0.001 vs. control; Figure 5B).

### Cell Susceptibility to Immunosuppressants

**Figure 6:**
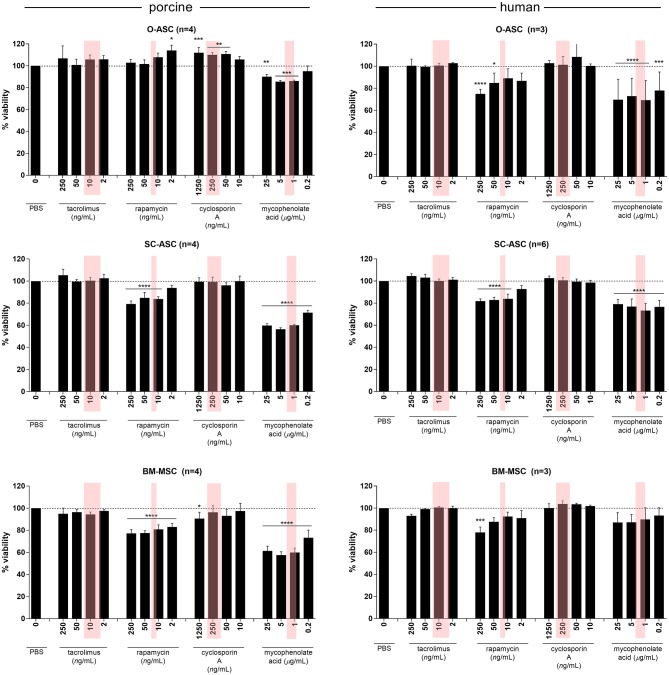
Susceptibility of MSCs to immunosuppressive agents. The three cell lines (O-ASC, SC-ASC, and BM-MSC) were exposed to 4 different immunosuppressive agents commonly used in transplantation for 36 h to assess the effect on cell proliferation using AlamarBlue. Expressed in % viable cells compared to control (no drug = vehicle+medium only). Error bars = SD. Red boxes indicate the approximate therapeutic ranges (Tacrolimus: 5–20 ng/mL, Rapamycin 16–24 ng/mL, Cyclosporin A 100–400 ng/mL, Mycophenolic Acid 1-3.5 ug/L). Two-way ANOVA with Tukey post-test *p < 0.05, **p < 0.01, ***p < 0.001, ****p < 0.0001 vs. control (PBS only).

All cell types from swine and human donors showed no significant differences in proliferation with Tac compared to controls at any concentration. A similar picture was found with CsA, but pO-ASC revealed a slight, yet significant higher proliferation starting from 50 ng/mL upwards (110.80 ± 2.3%, *p* < 0.01 vs. PBS; Figure 6).

In co-cultures with Rapa, however, there was a differential effect on cell proliferation: while porcine O-ASCs were not affected and human BM-MSCs only affected at high doses (87.55 ± 3.77 at 50 and 77.95 ± 4.95% at 250 ng/mL), pSC-ASC and pBM-MSC revealed diminished proliferation starting from Rapa doses of 10 ng/mL (83.74 ± 2.13% *p* < 0.0001 vs. PBS) and 2 ng/mL (82.97 ± 3.23% *p* < 0.0001 vs. PBS), respectively, which is including the therapeutic range. Similarly, in hO-ASC proliferation decreased already from 2 ng/mL (86.73 ± 7.06%), reaching significance at 50 ng/mL (84.80 ± 8.99% *p* < 0.05 vs. PBS); in hSC-ASC accordingly, there was a disturbing effect on proliferation from 10 ng/mL (83.88 ± 4.22% *p* < 0.0001 vs. PBS). The most striking effect was seen in co-cultures with MPA: all cell types were strongly affected already at doses of 0.2 ug/mL, including therapeutic doses (pO-ASC 86.21 ± 0.80% 1 ng/mL *p* < 0.001; pSC-ASC 71.35 ± 2.21%, pBM-MSC 73.27 ± 6.83% and hSC-ASC 76.54 ± 5.99% 0.2 ug/mL *p* < 0.0001; hO-ASC 77.95 ± 16.79% 0.2 ug/mL *p* < 0.001; all vs. PBS). Only human BM-MSCs were less affected and showed a trend for lower proliferation but not as much as the other cell types.

### Susceptibility of Cell Immunomodulatory Function to Immunosuppressants

**Figure 7:**
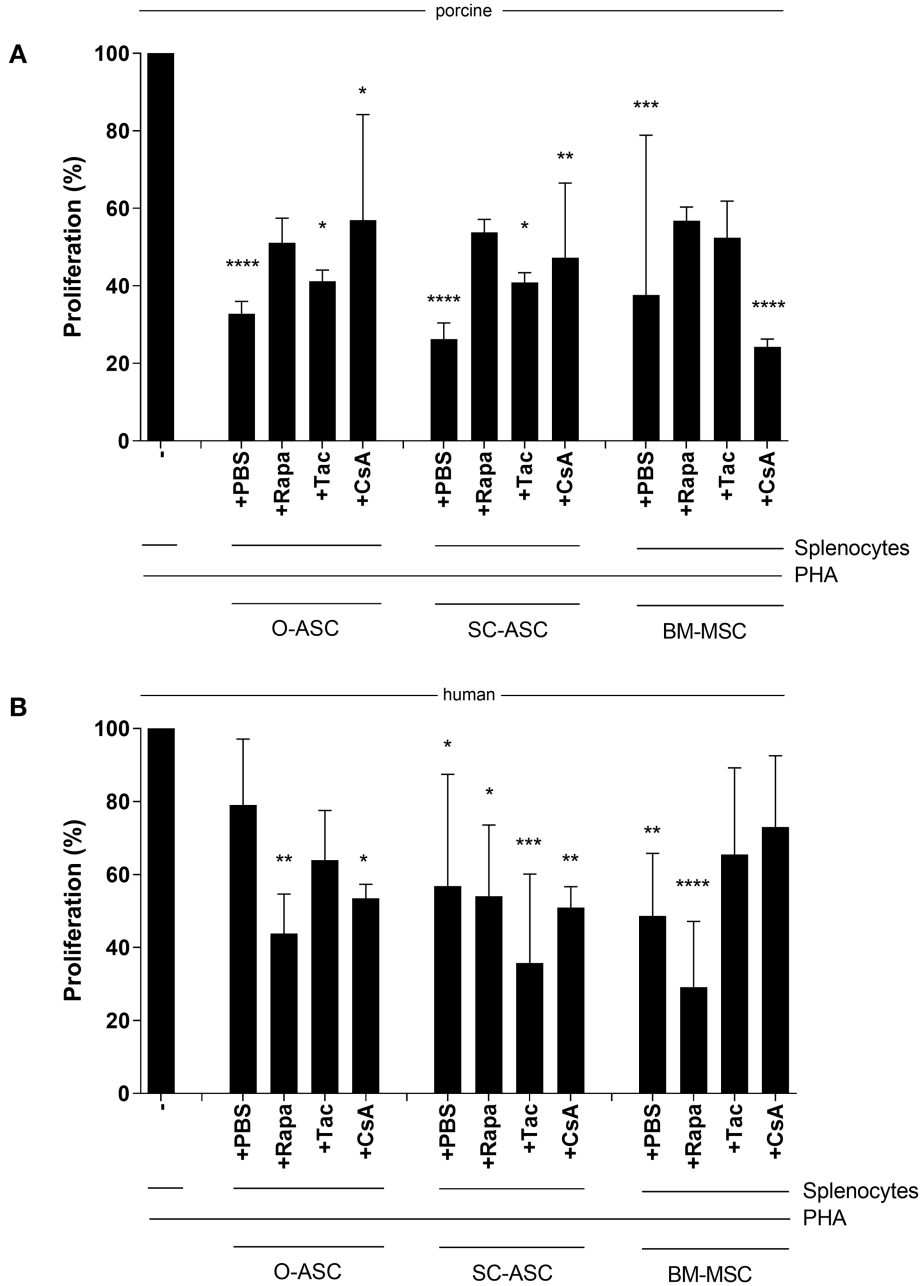
Susceptibility of MSC immunosuppressive function to immunosuppressive agents. Mixed lymphocyte reaction assays (MLR) were performed to assess the immunomodulatory potential of the three cell lines (O-ASC, SC-ASC, and BM-MSC) head-to-head of both porcine **(A)** and human **(B)** origin after incubation with immunosuppressive agents at therapeutic dosage (Tacrolimus 10 ng/mL, Rapamycin 10 ng/mL, Cyclosporin A 250 ng/mL) for 36 h. Yorkshire splenocytes were stimulated with PHA and co-cultured with the different cell lines at a 4:1 ratio. Expressed as percentage proliferation in relation to positive control. Error bars = SD. Two-way ANOVA with Tukey post-test. *p < 0.05, **p < 0.01, ***p < 0.001, ****p < 0.0001 vs. control (splenocytes+PHA).

ASCs' and BM-MSCs' immunomodulatory function was not significantly reduced in porcine cells when under the influence of Tac, Rapa or CsA compared to PBS control. There was, however, a minor reduction in suppression of allo-reaction with Tac (O-ASC 41.21 ± 2.86% vs. PBS 32.82 ± 3.18%; SC-ASC 40.86 ± 2.53% vs. 26.28 ± 4.15% PBS; BM-MSC 52.36 ± 9.48% vs. 37.65 ± 41.23% PBS) and Rapa (O-ASC 51.09 ± 6.37%, SC-ASC 53.81 ± 3.35%; BM-MSC 56.76 ± 3.59%) in all groups and with CsA only in O-ASC (56.96 ± 27.21%) and SC-ASC (47.22 ± 19.26%)(Figure 7A).

For human cells there were similar results with no significant alteration of the immunomodulatory ability of ASCs and BM-MSCs when co-cultured with the immunosuppressants. In hO-ASC proliferation was 63.95 ± 13.56% with Tac, 43.77 ± 10.87% with Rapa and 53.48 ± 3.81% with CsA vs. 79.00 ± 18.10% with PBS (Figure 7B); in hSC-ASC it was 35.69 ± 24.41% with Tac, 54.05 ± 19.47% with Rapa and 50.92 ± 5.71% with CsA vs. 56.84 ± 30.62% PBS. Finally, in hBM-MSCs proliferation was 65.43 ± 23.77% with Tac, 29.14 ± 17.96% with Rapa, 72.98 ± 19.54% with CsA vs. 48.63 ± 17.11% PBS.

## Discussion

This study is unique in that it evaluated the potential of MSCs for cytotherapies from three different anatomical donor sites from both swine und human origin after rapid culture-expansion with endothelial growth medium (EGM). As main finding, there was no significant difference between the three cell types in terms of immunomodulatory function or susceptibility to immunosuppressive agents, making them all ideal candidates for transplant-related cytotherapies in conjunction with immunosuppressive agents at usual therapeutic ranges. However, there are some advantages from an isolation, cultivation and proliferative point of view for the omental and, to a less extent, subcutaneous counterparts, which might favor their use, even more so when accounting for ease of harvest from these donor sites with low morbidity. The uniqueness of the present study is seen also in the direct comparison of the different cell lines between different species: the results suggest that immunomodulatory function and cell susceptibility to immunosuppressants found in swine are similar to that of human cells, enabling extrapolation of results from large animal studies to clinical application.

We used a rapid culture-expansion protocol to achieve high cell yields in short culture time as proposed earlier by Suga et al. (23). The use of EGM has not been previously published with the three cell lines used in our study compared head-to-head: our results suggest that *in vitro* MSCs from the three sources retain typical surface marker phenotypes, differentiation potential and immunomodulatory function under culture with EGM. Depending on the application, the need for MSCs in high numbers involves culture-expansion after cell isolation, prior to administration to recipient. In the scenario of transplant-related tolerogenic regimens, an early and repeated cell therapy with MSCs from the same deceased tissue donor as the transplanted organ might warrant better results (7, 25). Thus, rapid cell expansion is key after tissues harvest and cell isolation from the donors with genetic stability of the cells.

The use of omentum-derived ASCs in conjunction with subcutaneous ASCs for transplant related purposes has been proposed and investigated by our group previously (26). From a translational aspect, the use of both SC- and O-ASCs warrant increased cell yields and allow for high dosed cell therapy, while acting synergistically in their immunomodulatory function.

Isolation yields in the O-ASC group were significantly higher from porcine and human tissues compared to SC-ASC, even though the amount of tissue harvested was usually less: this is probably due to the loose nature of the omentum majus, which makes disruption by the enzymatic solution more effective. In general, there was high variability in the number of isolated cells per volume from the omentum. Yields and viability at the first passage were higher in O-ASC and SC-ASC, suggesting that for BM-MSCs the actual isolation protocol is not able to eliminate non-MSC cells adequately during isolation and plating. Cell viability throughout cultivation to P7 was lower in BM-MSC, significantly in human cells, under rapid expansion with EGM.

In a study by Toyoda et al., isolation of human ASCs from omentum majus and subcutis yielded fewer and more cells, respectively, compared to our study, probably due to the differing isolation protocol (12). As far it concerns pig-derived cells, Calle et al. found that ASCs from abdominal fat have longer PDT than their SC counterpart under culture with DMEM (27), which is in contrast to similar PDT in O-ASC and SC-ASC in our study under EGM. Other reports showed that BM-MSC had higher PDT than ASCs in the past (28–30), which was confirmed only in the first passage in our study.

Presence of FGF-2, as in the EGM, has been shown to promote ASC proliferation (31). Yoshimura et al. proposed ASC expansion with EGM and compared it to standard medium, D-MEM, in terms of multipotency and proliferation and found rapid growth and multilineage differentiation potential under EGM cultivation (23). They also suggested that FGF-2 might be crucial for MSC self-renewal. While for the first passage they demonstrated lower PDT, other than that we found similar doubling times in the following passages for all three cell types. Of note, porcine BM-MSC had the longest time to first passage requiring almost 100 h to double the cell population, followed by human BM-MSCs; however, in the following passages there was no significant difference compared to other cell types. Another study confirmed similar surface marker phenotype with EGM comparable to standard cultivation media, whereas the *in vivo* engraftment ability to the perivascular niche was increased with EGM (32). MSCs in that study did not show an increase in CD31 expression, suggesting that they did not differentiate in alternative lineages up to passage 3 or 4 under EGM. We found similar surface marker phenotype profiles as other groups (11, 27, 28, 30, 33) and there was no relevant difference between the cells. CD73 is known for lack of cross-reactivity of the CD73 antibody in pigs and was confirmed here (16, 34).

Although some authors reported similar adipogenic differentiation potential in bone marrow and adipose tissue derived MSCs (11), we found significantly increased differentiation potential for human O-ASC and SC-ASC compared to BM-MSC, as also found in other studies (35). In pig cells, however, the difference was subtle, while there was no significant difference between O-ASC and SC-ASC, even though other groups found higher affinity to adipogenic differentiation in SC-ASC (12). Another study comparing three different types of human ASCs found increased osteogenic differentiation by omentum-derived cells, while subcutaneous and pericardial derived cells had higher adipogenic potential (14). In their study, the authors found also longer PDT in cells from the pericardial niche. We did not explore chondrogenic and osteogenic differentiation potential because we focused on the immunomodulatory function and susceptibility of the cells to immunosuppressants in this study: however, trilineage differentiation potential has been shown in the past for the same MSC types with similar surface marker phenotype for both porcine and human-derived cells (14, 28, 29, 36).

There is a paucity of reports on the cell size for MSCs, especially comparing different cell types. Our analysis revealed slightly larger cell diameters in human MSCs compared to porcine ones, especially for BM-MSCs. We could not identify differences between the anatomical locations. Previously our group analyzed detached rodent ASCs and MSCs and found similar sizes for ASCs but larger diameters for BM-MSCs, which might be due to inter-species differences or different culture medium (24). Cell size is relevant mostly after intravascular, usually intravenous, administration because of potential entrapment of the cells in capillaries of organs such as the lung, spleen and liver, which might reduce the amount of MSCs reaching the target organ (37, 38). However, we did not further investigate this in our study.

We assessed the immunomodulatory function of MSCs from the different anatomical locations *in vitro* and found no relevant inter-group difference in human and porcine cells, while all cells exhibited dose-dependent suppression. To reach a relevant suppressive function on activated splenocytes, at least a splenocyte to MSC ratio of 8:1 was necessary for porcine ASCs, and 8:1 for human ASCs. Li et al. found similar suppressive effects between BM-MSCs and ASCs in MLRs with PHA activation (28), which is in line with other groups (33) and our results. Another group found that ASCs had higher immunomodulatory capacity in MLRs with peripheral blood mononuclear cells when compared to their BM counterpart (39). In general, in that study they found much higher suppressive capacity compared to our data, which might be due to isolation and culture differences, or the different MLR setup (peripheral blood mononuclear cells vs. splenocytes, CD3/CD28 activation against PHA). A study comparing human vs. porcine BM-MSCs for cardiac allotransplantation could show similar immunomodulatory action *in vitro* and *in vivo* (16).

The influence of immunosuppressive medications on MSCs has been investigated previously: Hoogduijn et al. found that MPA and Rapa inhibit MSC proliferation at therapeutic doses. We were able to confirm these results for pig and human MSCs. While pre-incubation of MSCs with Tac revealed an increased immunosuppressive function according to Hoogduijn et al. (18), we could not detect a relevant influence of the investigated MSCs on immunomodulatory function. Moreover, similar to our results, the same group found no difference in suppressive function between human BM-MSCs and ASCs *in vitro* and *in vivo* in a humanized mouse model (40). Putative advantages of using CsA in conjunction with MSCs for immunomodulation as suggested previously, could not be substantiated *in vitro* in our study (19, 20). CsA has also been used successfully with porcine ASCs in studies assessing their therapeutic effect in cardial infarction (41). In the toxicity assays MSCs were afflicted by reduced proliferation when incubated with Rapa and MPA, while Tac and CsA did not impair cell growth. Interestingly, O-ASC of porcine origin and human BM-MSC were less susceptible to Rapa and MPA, respectively. In previous own *in vitro* studies, we found that anti-lymphocyte serum and Tac both dose-dependently affected rodent ASC and MSC viability and proliferation already at clinically relevant doses (24).

To investigate whether MSCs are influenced by the immunosuppressants in their immunomodulatory functionality, we incubated the cells with Tac, Rapa and CsA at a clinically relevant dose in MLRs (MPA was excluded according to toxicity assay results). We found no significant impact of the different drugs on MSCs, but there was a non-significant trend showing slight worse suppressive action of porcine MSCs under immunosuppressant influence, while for human cells this was not the case.

While the cell isolation method applied in this study was not performed under a specific Food and Drug Administration approved protocol, it is analogous to the process employed in commercially available closed systems for cell isolation and has been shown by our group to result in a similar cell product as have been used in our own FDA sanctioned clinical trials (42, 43). Furthermore, as cultivation and expansion is concerned, we followed a strict, standardized and reproducible protocol, warranting future translation to regulatory conformity and similar to the type of expansion protocols used in cell therapy clinical trials (44).

In summary, MSCs from omentum, subcutaneous tissues, and bone marrow of both human and porcine origin share similar behavior in terms of surface marker phenotype, immunomodulatory function and susceptibility to immunosuppressants. However, the data show superiority of ASCs in isolation yields, viability and potential for rapid culture-expansion. EGM culture expansion seems to retain the functionality of both porcine and human MSCs, making is a useful tool for cell therapy studies in which the necessary cell dose exceeds the quantity of cells harvested.

## Data Availability Statement

The raw data supporting the conclusions of this article will be made available by the authors, without undue reservation, to any qualified researcher.

## Ethics Statement

Deceased tissue donors were referred after informed consent by the Center for Organ Recovery and Education (CORE).

## Author Contributions

RS: conception and design, financial support, collection and/or assembly of data, data analysis and interpretation, manuscript writing. MW: provision of study material, collection and/or assembly of data, data analysis and interpretation. SO and CK: provision of study material, collection and/or assembly of data. WZ: collection and/or assembly of data, data analysis and interpretation. JP: data analysis and interpretation, manuscript writing. VG: conception and design, provision of study material, data analysis and interpretation. MS: conception and design, data analysis, and interpretation. LK: collection and/or assembly of data, data analysis and interpretation, and manuscript writing. KM: conception and design, provision of study material, and data analysis and interpretation. JR: conception and design, financial support, provision of study material, data analysis and interpretation, manuscript writing, and final approval of manuscript.

## Conflict of Interest

The authors declare that the research was conducted in the absence of any commercial or financial relationships that could be construed as a potential conflict of interest.

## Abbreviations

ASC: adipose-derived mesenchymal stem cell
BM-MSC: bone marrow MSC
CsA: cyclosporin A
IS: systemic immunosuppression
MLR: mixed lymphocyte reaction assay
MPA: mycophenolic acid
MSC: mesenchymal stem (stromal) cell
O-ASC: omental ASC
PDT: population doubling time
Rapa: rapamycin
RT: room temperature
SC-ASC: subcutaneous ASC
SVF: Stromal vascular fraction
Tac: tacrolimus
WBC: white blood cells.
